# Should We Routinely Exclude Retroperitoneal Abscess in Cases of Hip Periprosthetic Joint Infections?

**DOI:** 10.7759/cureus.15126

**Published:** 2021-05-19

**Authors:** Efstratios D Athanaselis, Fotios Papageorgiou, Nikolaos Stefanou, Theofilos Karachalios, Socratis Varitimidis

**Affiliations:** 1 Department of Orthopaedic Surgery and Musculoskeletal Trauma, University Hospital of Larissa, Larissa, GRC

**Keywords:** periprosthetic joint infection, retroperitoneal abscess, iliopsoas abscess, total hip arthroplasty, 2-stage hip arthroplasty revision

## Abstract

Hip periprosthetic joint infections (PJIs) with concomitant retroperitoneal abscesses may not be common clinical situations but they can be easily misdiagnosed affecting the effectiveness of infection control and eradication interventions. We present the case of a 75-year-old female patient with a late hip PJI complicated with iliopsoas abscess that was barely discovered intraoperatively. Literature review supports our recommendation of a high index of suspicion in cases of hip PJI and even routinely imaging examination of pelvis and abdomen for retroperitoneal involvement exclusion.

## Introduction

Retroperitoneal abscesses represent uncommon clinical entities, being diagnosed either solitarily or secondarily, in association with intra- or extra-abdominal infections. A well-known anatomical communication exists between the hip and retroperitoneum due to anatomical communicating spaces and characteristic course and insertion of the iliopsoas muscle. This anatomical relationship leads to hip-related symptoms due to retroperitoneal etiology and vice versa, and combined infections of the retroperitoneal space and the native or prosthetic hip joint, as well. Periprosthetic Joint Infections (PJI’s) are possibly devastating complications, resulting in considerable morbidity and mortality apart from significant financial burden [1].

Starting from a case study concerning a late hip PJI treated by two-stage hip revision arthroplasty, a literature review of concomitant periprosthetic hip joint infections and retroperitoneal abscesses is presented here.

## Case presentation

A 75-year-old female patient was admitted to our department due to persistent pain in her right hip, with acute onset a week before and progressive deterioration, making the patient unable to bear weight on the symptomatic side. Her medical history included diabetes mellitus and hypertension, but no neoplasm or immunosuppressant therapy. The patient had been operated on for a total arthroplasty on the right hip five years ago but no relevant functional impairment or clinical symptom of hip arthroplasty during the five-year postoperative period was referred.

Clinical examination revealed painful and restricted hip joint motion, swelling of the proximal thigh, and mild fever (<38°C). There was no surgical site or sinus tract. The patient was in good general medical condition, without further clinical complaints other than the right hip pain. No history of recent pneumonia, upper respiratory tract infection (URTI), or urinary tract infection (UTI) was referred. However, laboratory workup revealed excessive leukocytosis (white blood cells [WBC] 43800/μl, neutrophil 94.1%), C-reactive protein 28.42 mg/dl, erythrocyte sedimentation rate (ESR) 143 mm/h, and blood glucose 218.2 mg/dl. A thorough examination of possible adjacent or distant sites that could have caused a hematogenous spread of infection in the hip or retroperitoneal space (i.e. echocardiography for bacterial endocarditis) turned out to be negative.

**Figure 1:**
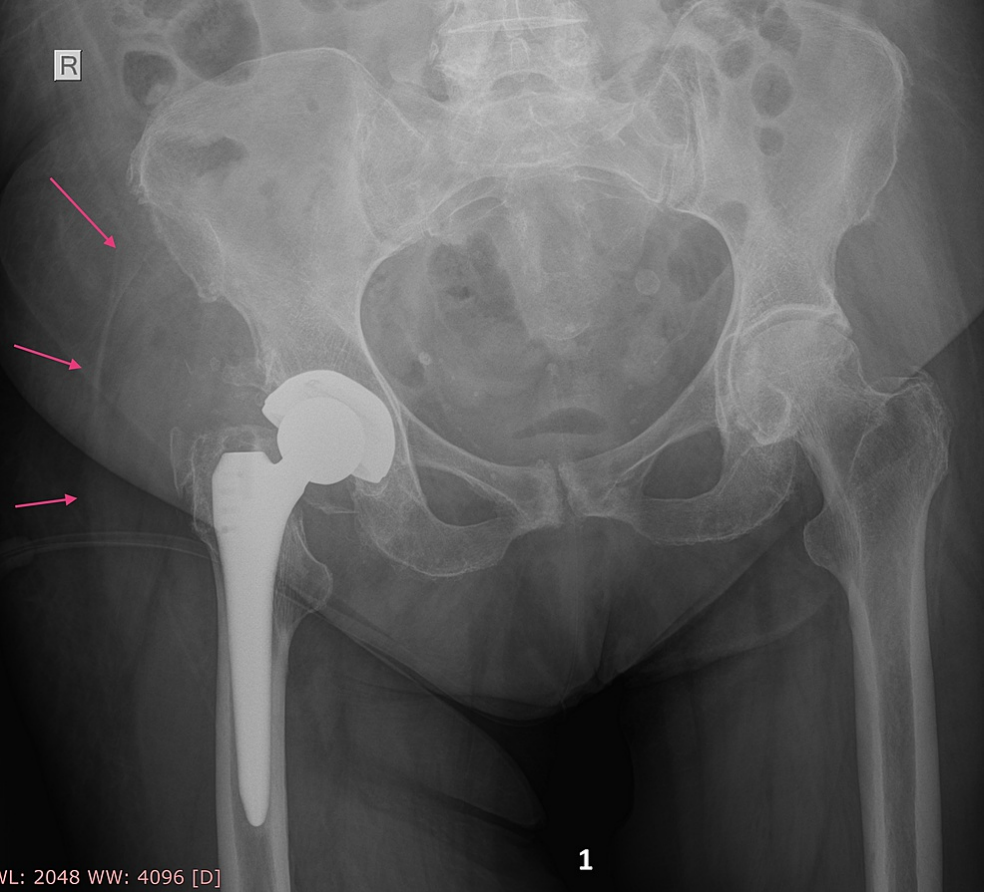
Pro-operative X-ray. Pro-operative X-ray of pelvis and right hip total arthroplasty reveals hip space effusion (red arrows) but there is no clear sign of iliopsoas abscess.

A pelvic X-ray revealed effusion around the hip prosthesis. Though common X-ray is not a great imaging study to rule out an abscess, subtle signs of iliopsoas abscess presence (increased radiodensity in the area of right iliopsoas muscle) were misregarded (Figure 1). Therefore, further imaging examination (CT scan, positron emission tomography-computed tomography/PET CT or MRI) of the pelvis that could have revealed retroperitoneal space effusion was not obtained. Triplex ultrasound examination of the lower limbs (due to thigh swelling) revealed an ipsilateral femoral vein thrombosis.

Hip joint aspiration revealed purulent fluid under pressure and the patient was driven towards operative treatment on an urgent basis. Surgical drainage via posterior hip approach was performed draining more than one liter of pus. Intra-operatively, an additional purulent effusion was noticed again after the hip evacuation, irrigation, and antibiotic cement spacer placing. Meticulous investigation of pus origin drove to the lesser trochanteric region. Blunt dissection via iliopsoas insertion evacuated an iliopsoas abscess. Decompression and drainage of the communicating spaces were deemed sufficient, and therefore, no additional approach was needed. 

The first stage of hip PJI was successful as it included complete pus drainage, removal of implants (that were not loose), thorough wash-out, and antibiotic-loaded (with vancomycin 2gr, gentamicin 1gr, clindamycin 1gr) cement spacers application according to the department’s PJI treatment protocol. Intra-operative, pus and tissue culture samples were acquired (six in total) and two drains were left in the surgical wound (one in hip joint space and one in subcutaneous space).

**Figure 2:**
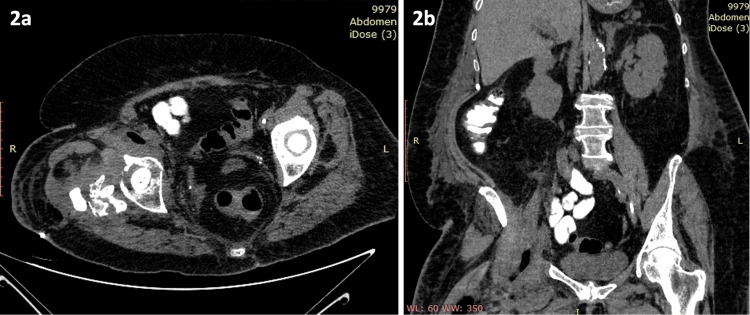
Postoperative CT scan. Transverse (2a) and coronal (2b) views of postoperative CT scan showing various empty spaces in retroperitoneum and hip region created by abscesses drainage.

Immediate and significant clinical and laboratory improvement of the patient was observed after the surgical debridement. Post-operative CT scan of the abdomen (including lower spine) and pelvis revealed multiple empty spaces at the retroperitoneal and hip region, indicative of evacuated abscesses and confirming iliopsoas involvement (Figures 2a, 2b). 

**Figure 3:**
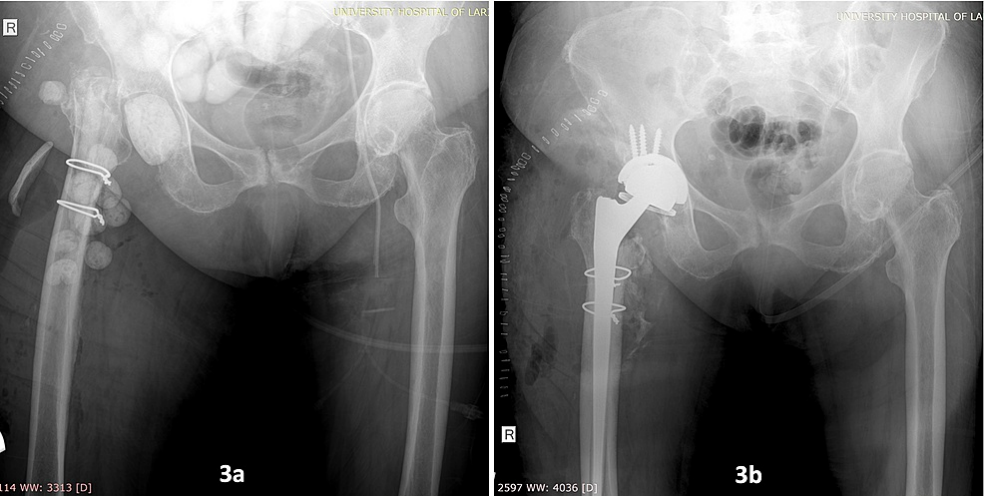
Postoperative X-rays. X-ray after first-stage surgical debridement and antibiotic cement spacers application (3a) and after second-stage reimplantation with constrained total hip arthroplasty (3b).

Cultures of pus and tissue samples, and the removed hardware as well, revealed *Streptococcus pneumoniae* and *Staphylococcus epidermidis*, and targeted antibiotics were given for six weeks (intravenously for the first three weeks and per os afterward). Meanwhile, additional surgical debridement took place twice on the occasion of antibiotic cement spacers replacement and removal procedures) (Figure 3a).

The overall hospital stay was 24 days; the patient then was closely followed up on an outpatients’ basis afterward, including clinical and laboratory examination (Figure 3b).

The postoperative period was uneventful, without any major systematic or local complications or need of rehospitalization. A total of 12 weeks later, infection was considered successfully treated on the basis of negative clinical examination, improved laboratory findings (WBC 7200/μl, C-reactive protein 7.56 mg/dl, ESR 43 mm/h, blood glucose 109 mg/dl, and negative blood cultures), and a new CT scan without signs of infection recurrence. Therefore, we proceeded to the second stage of revision hip arthroplasty by the use of a constraint total hip arthroplasty and further antibiotic coverage for four weeks. During the 12 months of follow-up, the patient is free of symptoms and fully active.

## Discussion

Retroperitoneal abscesses can be present either primarily or secondarily. Primary abscesses are believed to be mainly due to hematogenous or lymphatic spread, usually have insidious onset with intermittent fever, fatigue, and moderate back and limp pain. Mild clinical symptoms are responsible for delayed initial diagnosis, especially in younger patients. Staphylococcus aureus is most often the pathogenic microorganism cultured.

Primary psoas abscess is often associated with acne in the region. On the other hand, secondary abscesses originate from a nearby retroperitoneal infection, a spinal, intra-abdominal, or urogenital one. Traditionally, psoas abscess is a condition associated with tuberculosis of the spine. Given that tuberculosis complications have become rare nowadays, intra-abdominal infection is the most common cause of secondary psoas abscess. Among various causes like Crohn's disease, ulcerative colitis, diverticulitis, perforated appendix, renal or perirenal infection, malignancy, spinal osteomyelitis, or postoperative infections, Tabrizian et al. found that inﬂammatory bowel disease is the most common cause of psoas abscess. Sporadically, there are reports of colon or duodenum perforation causing a retroperitoneal abscess or even intramuscular pharmaceutical injections in the region [2-4].

Common symptoms are the triad of pain in the lumbar area, pelvis, or thigh, hip flexion deformity, and fever. Chills, weight loss, or localized pain are not uncommon clinical characteristics. Pain is generally increased by hip extension or hyperextension on the involved side due to psoas muscle stretch [5, 6].

However, there is limited literature via small case series or case reports concerning such infections associated with PJI septic arthritis or periprosthetic hip infection even though anatomic communication between hip joint and retroperitoneal space does exist. Different anatomic pathways have been described: via greater or lesser sciatic foramen, the obturator or femoral canal, along with the fascial coverings of the iliacus muscle to its insertion at the lesser trochanter, and through the iliopsoas bursa which is present in 14% of patients. This bursa communicates with the native hip joint and is attached to the pseudocapsule formed postoperatively after hip arthroplasty [7-10].

Retroperitoneal abscess due to ascending expansion of primary septic arthritis of the hip is rare, mainly seen in children but unfortunately misdiagnosed. Diagnostic delays can lead to life-threatening sepsis with considerably higher morbidity and mortality. Moreover, retroperitoneal abscesses associated with hip PJI’s have an incidence of 1-2%, and though sporadically referred to in the literature, successful drainage has great importance in infection eradication [11, 12].

Besides the previously reported possible communication pathways between retroperitoneal and hip joint space, PJI of the hip can spread to retroperitoneum via acetabular screws protruding iliac bone cortex, or even by anterior capsulorrhaphy performed during hip replacement. Studies have also revealed metal-on-metal bearing surfaces of hip arthroplasty as a predisposing factor for concomitant retroperitoneal infection due to local immunological alterations associated with adverse local tissue reaction [13-15].

Though an associated retroperitoneal abscess can complicate hip PJI eradication, current clinical data reveals this condition as under-diagnosed. The similarity of retroperitoneal abscess clinical symptoms to those of hip septic arthritis or PJI may confuse the diagnosis [16]. Additional strong reasons for misdiagnosing such an abscess are the inability of standard X-ray imaging to identify the presence of psoas abscesses (Figure 1), as well as the non-routinely use of CT scans in cases that hip aspiration confirms PJI [17]. Focusing on hip PJI and its two-stage treatment may hinder retroperitoneal abscess detection, sufficient drainage, and infection control despite continuous aggressive debridement and implant removal. 

In a study performed by Lawrenz et al., five cases with an analogous clinical scenario including co-existing hip periprosthetic infection and retroperitoneal abscess, are presented, and diagnostic and treatment challenges are discussed. A CT scan of the abdomen and pelvis with contrast is the standard imaging study in psoas abscess diagnosis taking into account that MRI is not quite helpful in PJIs. However, neither of these examinations are routinely recommended by MusculoSkeletal Infection Society (MSIS) consensus guidelines in the diagnosis of a PJI [18].

Dauchy et al. analyzed retrospectively 106 patients with hip periprosthetic infections using abdominal CT scans and recognized a 12% incidence of concomitant psoas abscess. Further analysis led the authors to recommend that a CT scan should be considered for patients presenting with hematogenous hip periprosthetic infections (delayed presentation with sudden onset of pain and fever) and for those having a past medical history of neoplasm. This approach leads to more strategic treatment plans in these cases [17].

Concerning infection’s origin in the case presented, hip PJI by *Streptococcus pneumoniae* is more than likely hematogenous but, this bacterium can also cause hematogenous spinal disease with abscess trickling down the iliopsoas and infecting the joint. However, in many cases with retroperitoneal abscess associated with hip PJI, it is not always easy to understand which site was infected first. Though well documented that late (>2 years postoperatively) hip arthroplasty infections with sudden onset are hematogenous and they can spread proximally to retroperitoneal space via anatomical pathways, a retroperitoneal abscess can also be the cause of hip PJI. In such cases, possible causes of iliopsoas abscess (mainly concerning the GI tract) should be investigated and treated.

In our case, the lack of clinical features other than hip pain, resulted in incomplete preoperative diagnostic procedures. However, psoas abscess was suspected due to purulent drainage from the lesser trochanteric region during the procedure and evacuated during surgical debridement of first-stage treatment of hip PJI. Failure to recognize it could lead to infection persistence, and patient’s destabilization due to sepsis as long as antibiotics would only suppress the infection rather than eradicating it. Moreover, bacteria persisting in the psoas abscess can cause infection relapse, infecting new prosthetic implants [17].

Various pathogens have been found to be responsible for retroperitoneal iliopsoas muscle abscesses with ipsilateral hip PJI. *Staphylococcus aureus* and *methicillin-susceptible Staphylococcus aureus (MSSA)*, *Streptococcus anginosus*, *Streptococcus group C*, and *Mycobacterium tuberculosis* seem to be common causes of infection but there are cases in which *Enterococcus faecalis*, *Propionibacterium acnes*, *Candida* and *Serratia marcescens* have been isolated [14, 16, 18-20].

Based on relevant clinical data and in this clinical scenario, it was appropriate to image the abdomen and retroperitoneal space. Imaging investigation of the lower abdomen and retroperitoneal space should be routinely conducted when approaching hip PJI even without obvious clinical features of retroperitoneal localization of the infection. Simple X-rays do not always reveal iliopsoas abscesses. In fact, signs may easily escape attention and high expertise and meticulous observation together with an awareness of this clinical condition, are determinants. CT scan, PET CT, and MRI (with low-signal intensity on T1 and relatively higher signal intensity on T2-weighted images) are definitely accurate and efficient diagnostic tools. However, ultrasound examination can also investigate retroperitoneum with the advantages of significantly lower cost, ease of performance, and of course, with no ionizing radiation exposure.

Orthopedic surgeons need to be aware of the possible concomitant presence of retroperitoneal space infection when dealing with late, hematogenous total hip arthroplasty infection. A high index of suspicion and retroperitoneal space imaging help in preoperative planning of hip PJI treatment [17, 18].

## Conclusions

Retroperitoneal abscesses associated with hip periprosthetic infections are often misdiagnosed as they are rare. Such abscesses can result from hip PJI expansion or the cause of PJI as well, especially in patients with GI pathology. Clinical symptoms and signs of PJI may overlap retroperitoneum involvement leading to insufficient surgical debridement and incomplete eradication of hip infection. Routine pelvis and abdomen imaging investigation or a high index of suspicion can reveal an additional space of pus formation that must be taken into account during first stage surgical debridement.
